# Supplementary material for: Impact of Glucose, Inflammation and Phytochemicals on ACE2, TMPRSS2 and Glucose Transporter Gene Expression in Human Intestinal Cells
Source: Antioxidants (Basel). 2025 Feb 21;14(3):253. doi: 10.3390/antiox14030253 (PMC11939507; doi:10.3390/antiox14030253)
Supplement: Supplementary file 1 [file antioxidants-14-00253-s001.zip › antioxidants-3405139-supplementary.pdf]

Supplementary Material

Table S1: The effect of selected phytochemicals and drugs on the expression of ACE2 and TMPRSS2

Compound/Extract	Dose (µM)	ACE2	TMPRSS2	Justification	References
**Phytochemicals**					
Punicalagin	50	NS	NS	Inhibit spike-ACE2 interaction; decrease ACE2 and TMPRSS2 expression	[1]
Quercetagetin	50	NS	NS	Inhibit SARS-CoV-2 propagation	[2]
Genistein	50	NS	↓	Anti-androgen; decrease TMPRSS2 expression	[3,4]
Sulforaphane	10	NS	↓	Decrease TMPRSS2 expression	[5]
Artemisinin	50	NS	↓	Inhibit SARS-CoV-2 propagation; anti-androgen	[6,7]
Resveratrol	50	NS	NS	Modulate ACE2 expression	[8]
Curcumin	50	NS	NS	Modulate ACE2 expression	[9]
Galangin	50	NS	NS	Inhibit ACE2	[10]
Apigenin	50	NS	↓	Potential ACE2 inhibitor; structural similarity to genistein	[11]
Daidzein	50	NS	↓	Structural similarity to genistein; decrease TMPRSS2 expression	[4]
Ursolic acid	50	NS	NS	Inhibits the main protease (M^{pro}) of SARS-CoV-2	[12]
Vitamin C	100	NS	NS	Used to treat the common cold	
**Drug**					
Dexamethasone	10	NS	↑	Drug candidate against COVID-19; anti-inflammatory	[13]
Apelin 13	10	NS	NS	Upregulate ACE2 expression	[14]
Estradiol		NS	NS	Modulate ACE2 and TMPRSS2 expression	[15,16]
Simvastatin	6	NS	NS	Modulate ACE2 expression	[17]

Enzalutamide	10	NS	NS	Anti-androgen	[18]
Vinclozolin	10	NS	NS	Anti-androgen	[19]
Pantoprazole	50	NS	NS	Increase ACE2 and TMPRSS2 expression	[20]
Nicotine	6	NS	NS	Smoking linked with increased ACE2 and TMPRRS2 expression in lung tissues	[21]
Vitamin D	6	NS	NS	Modulate ACE2 expression	[22]
Sodium selenate	5	NS	NS	Lower the risk and severity of COVID-19	[23]
**Plant extract**					
Green tea extract	1 mg/mL	NS	NS	Contains compounds that may bind to ACE2; modulate TMPRSS2 expression	[9,24]
Sugar cane extract	1 mg/mL	NS	NS	Source of unusual (poly)phenols	
Saponin Quillaja sp.	1 mg/mL	NS	NS	Decreased ACE activity, AngII concentration and ACE mRNA	[25]

NS - not significant; ↑ - significant upregulation; ↓ - significant downregulation; ACE - angiotensin-converting enzyme; ACE2 - angiotensin-converting enzyme 2; AngII - angiotensin II; TMPRSS2 - transmembrane serine protease 2.

Table S2: The effect of 4 h and 60 h genistein, apigenin, artemisinin and sulforaphane treatment on ACE2, SGLT1, GLUT2 and TMPRSS2 mRNA expression in standard and inflamed Caco-2/TC7 cells.

**Standard model**								
	**Treatment time - 4 h**				**Treatment time - 60 h**			
	**Genistein**	**Apigenin**	**Artemisinin**	**Sulforaphane**	**Genistein**	**Apigenin**	**Artemisinin**	**Sulforaphane**
**ACE2**	↓	ns	↑	ns	↓	↓	ns	↓
**SGLT1**	↓	ns	ns	ns	↓	↓	↓	ns
**GLUT2**	↓	↓	ns	↓	↓	↓	ns	↓
**TMPRSS2**	↓	ns	↓	ns	↓	↓	↓	↓
**Inflamed model**								
	**Treatment time - 4 h**				**Treatment time - 60 h**			
**ACE2**	↓	↑	↓	ns	↓	↑	↑	ns
**SGLT1**	↓	ns	↓	↓	↓	↑	↑	ns
**GLUT2**	↓	↓	↓	↓	↓	↓	ns	↓
**TMPRSS2**	↓	ns	↓	↓	↓	ns	↑	ns

ns - not significant; ↑ - increased expression; ↓ - decreased expression.

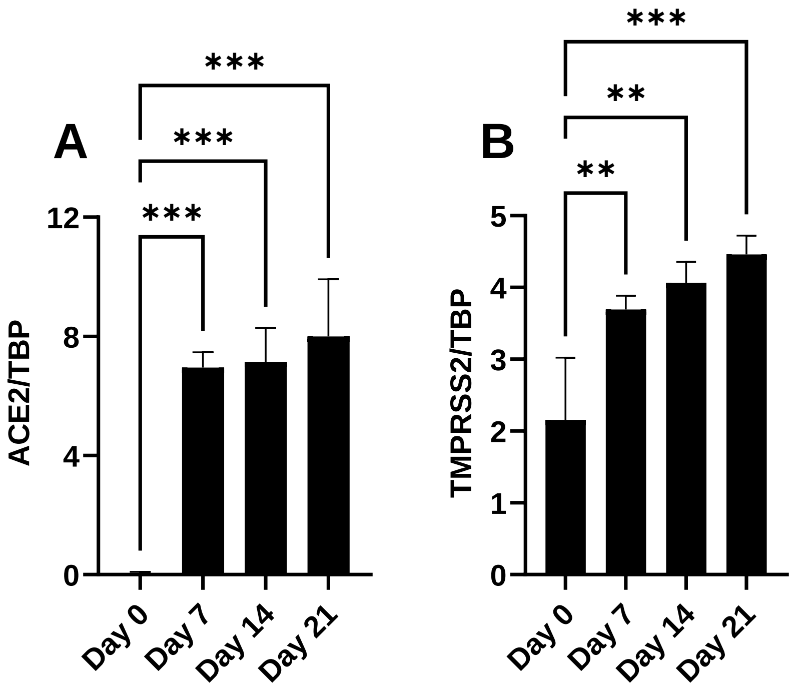

Figure S1: Caco-2/TC7 cell differentiation and ACE2 and TMPRSS2 gene expression. Change in ACE2 and TMPRSS2 mRNA levels in Caco-2/TC7 cells with differentiation. Total RNA was extracted, reverse transcribed and absolute copies of cDNA measured by ddPCR and expressed relative to reference gene TBP. Data are mean ± SD (n/N = 6/3). Significant differences determined by one-way ANOVA with Fisher's LSD multiple comparisons test. ** $P < 0.01$. *** $P < 0.001$.

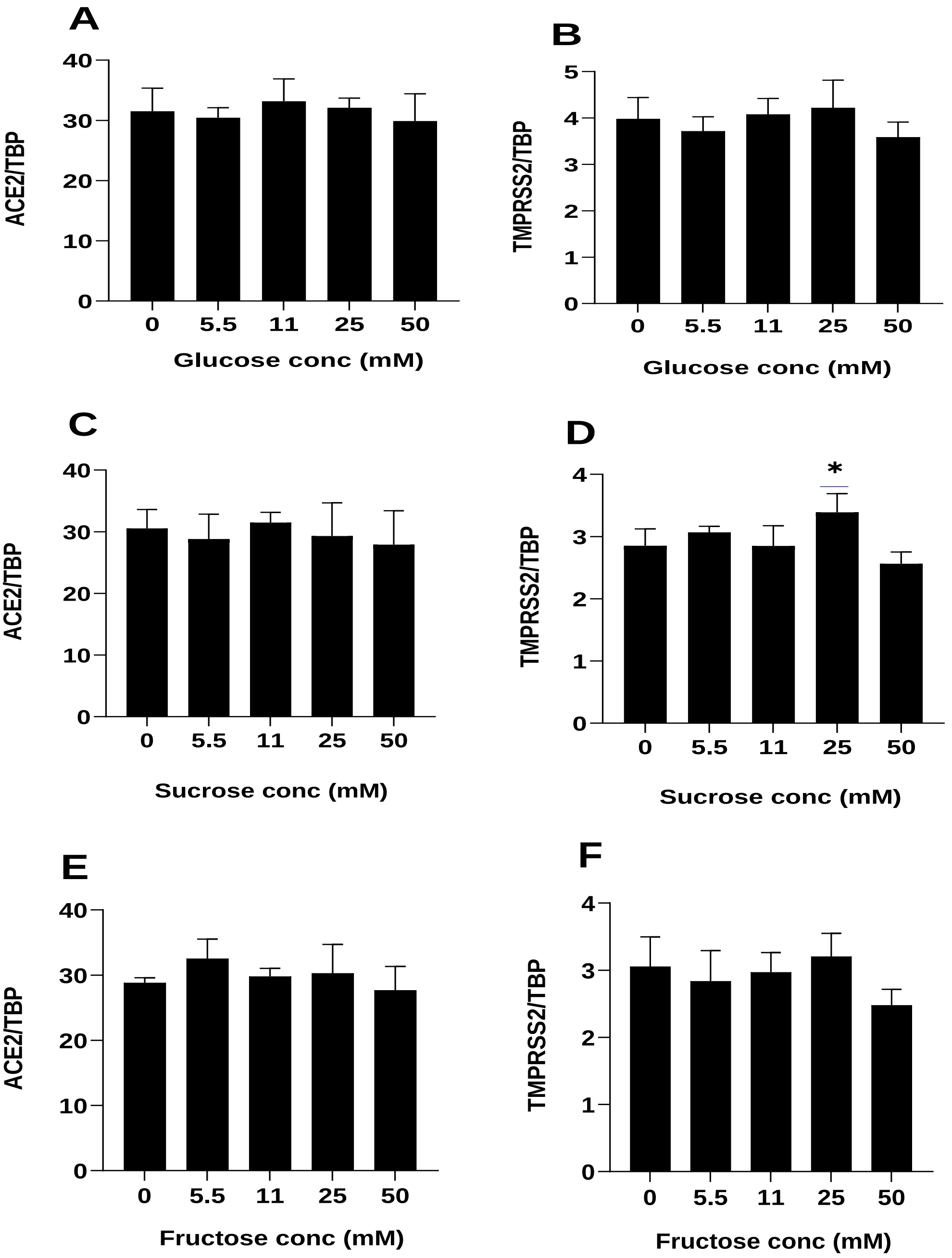

Figure S2: The effect of sugars on ACE2 and TMPRSS2 gene expression. The effect of glucose, sucrose and fructose on ACE2 (A, C, E) and TMPRSS2 (B, D, F) mRNA. Caco-2/TC7 cells differentiated for 21 days were exposed to the sugars (≤ 50 mM) for 4 h. Total RNA was extracted, reverse transcribed and absolute copies of cDNA measured by ddPCR and expressed relative to reference gene TBP. Data are mean ± SD (n/N = 6/3). Significant differences were determined by one-way ANOVA with Fisher's LSD multiple comparisons test; *$P < 0.05$.

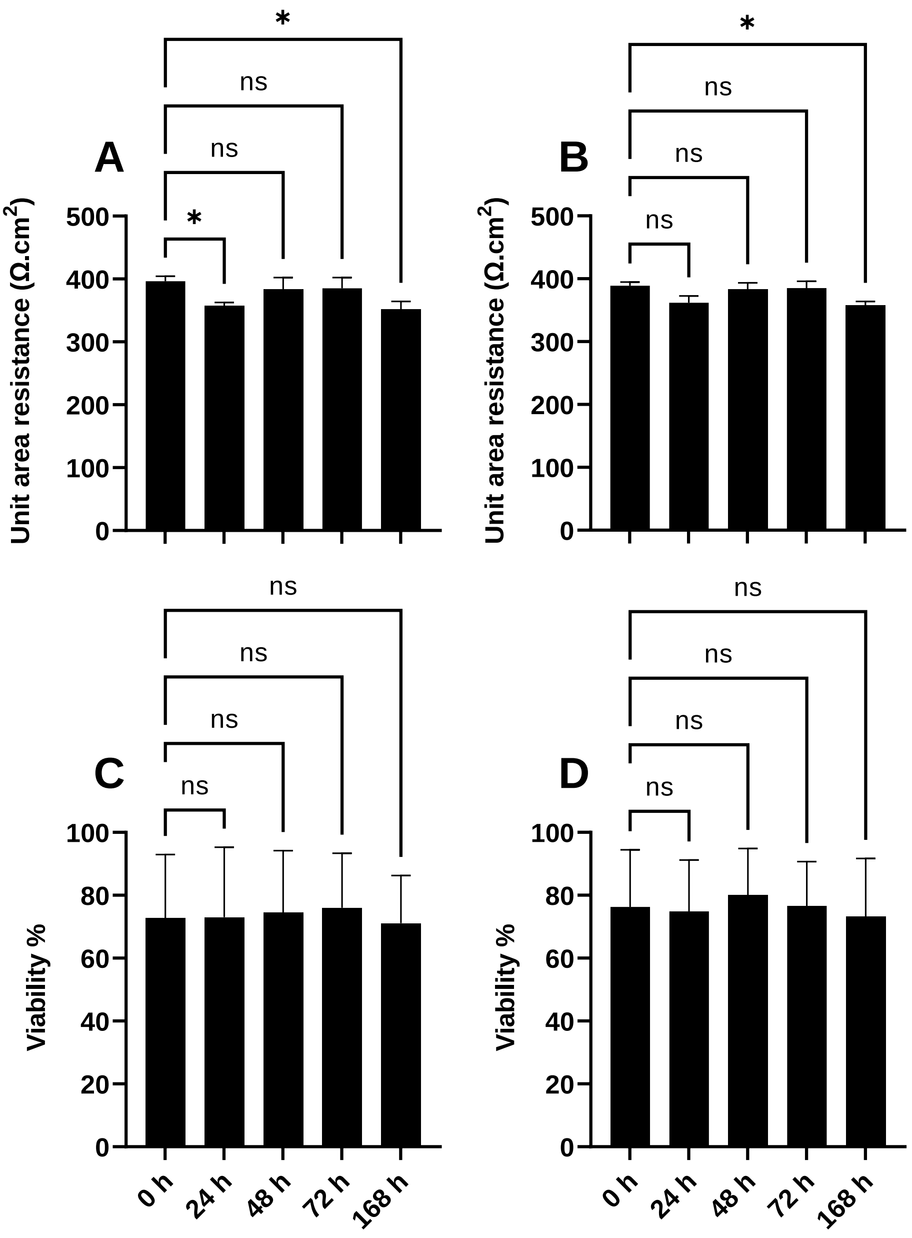

Figure S3: Effect of inflammation on Caco-2/TC7 cell monolayer integrity and cell viability. Transepithelial electrical resistance (TEER) values of cells grown in (A) 5.5 mM or (B) 25 mM glucose media, and viability (assessed by Trypan Blue exclusion) of cells grown in (C) 5.5 mM or (D) 25 mM glucose media treated with a cytokine cocktail containing IL-1β (25 ng/mL) and TNF-α (50 ng/mL) for ≤ 168 h. Data are expressed as mean ± SD (n/N = 6/3). Significant differences were determined by one-way ANOVA with Fisher's LSD multiple comparisons test; ns - not significant, $^*P < 0.05$.

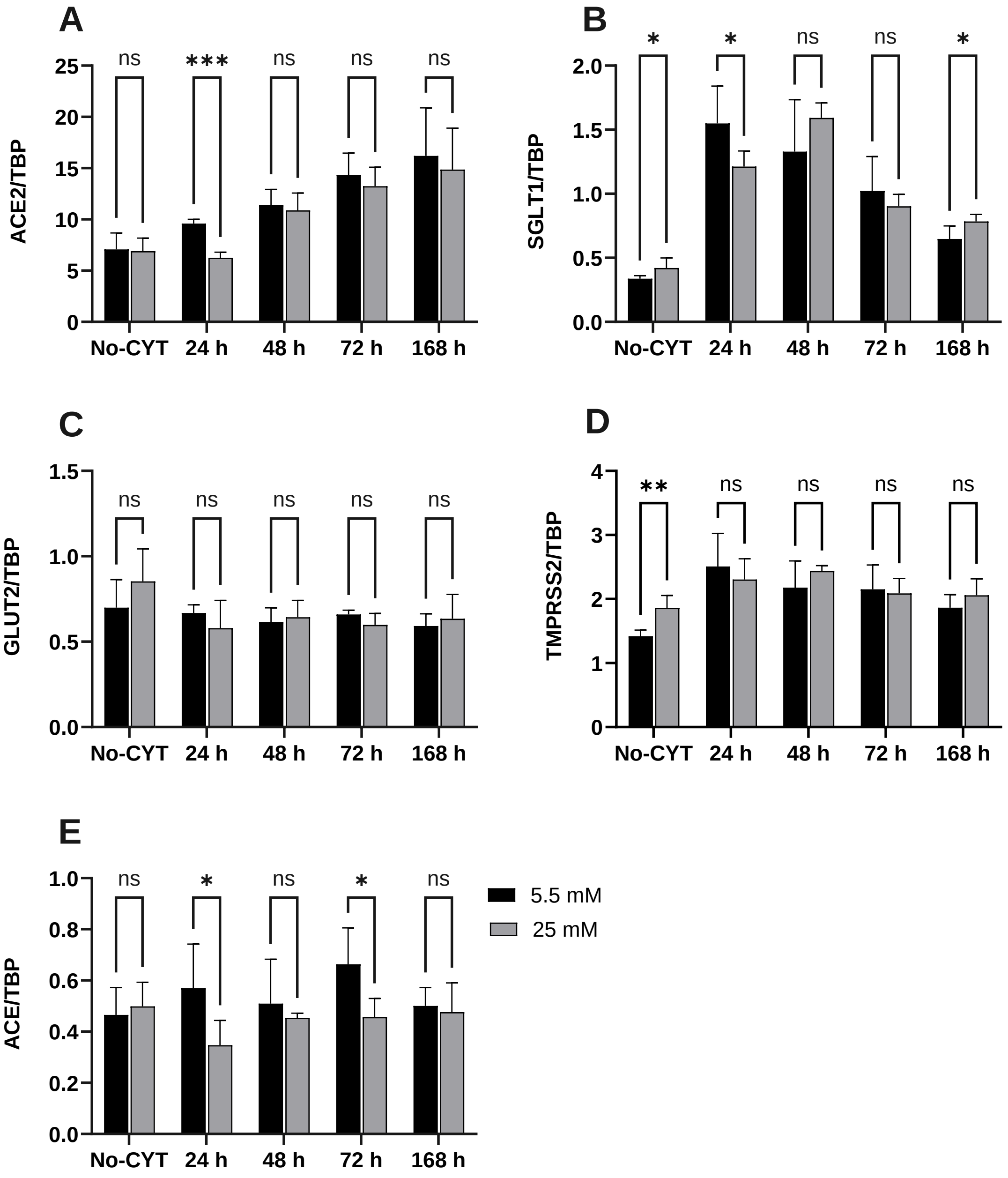

Figure S4: A comparison of the effects of pro-inflammatory cytokines on ACE2, SGLT1, GLUT2, TMPRSS2 and ACE mRNA in Caco-2/TC7 cells cultured in normal and high glucose. Cells were grown in 25 mM glucose for the first 7 days of differentiation and in either 5.5 mM (normal glucose) or 25 mM (high glucose) glucose for the final 7 days, until day 14. A cytokine cocktail containing IL-1β (25 ng/mL) and TNF-α (50 ng/mL) was added to the basolateral compartment from day 7, day 11, day 12 or day 13 (≤ 168 h). Total RNA was extracted on day 14, reverse transcribed and absolute copies of cDNA measured by ddPCR and

expressed relative to the reference gene TBP. Changes in expression of (A) ACE2, (B) SGLT1, (C) GLUT2, (D) TMPRSS2, and (E) ACE are presented. Data are mean ± SD (n/N = 6/3). No-CYT – control with no cytokine cocktail added. Significant differences were determined by two-way ANOVA with Fisher's LSD multiple comparisons test; ns – not significant, *$P < 0.05$, **$P < 0.01$, ***$P < 0.001$.

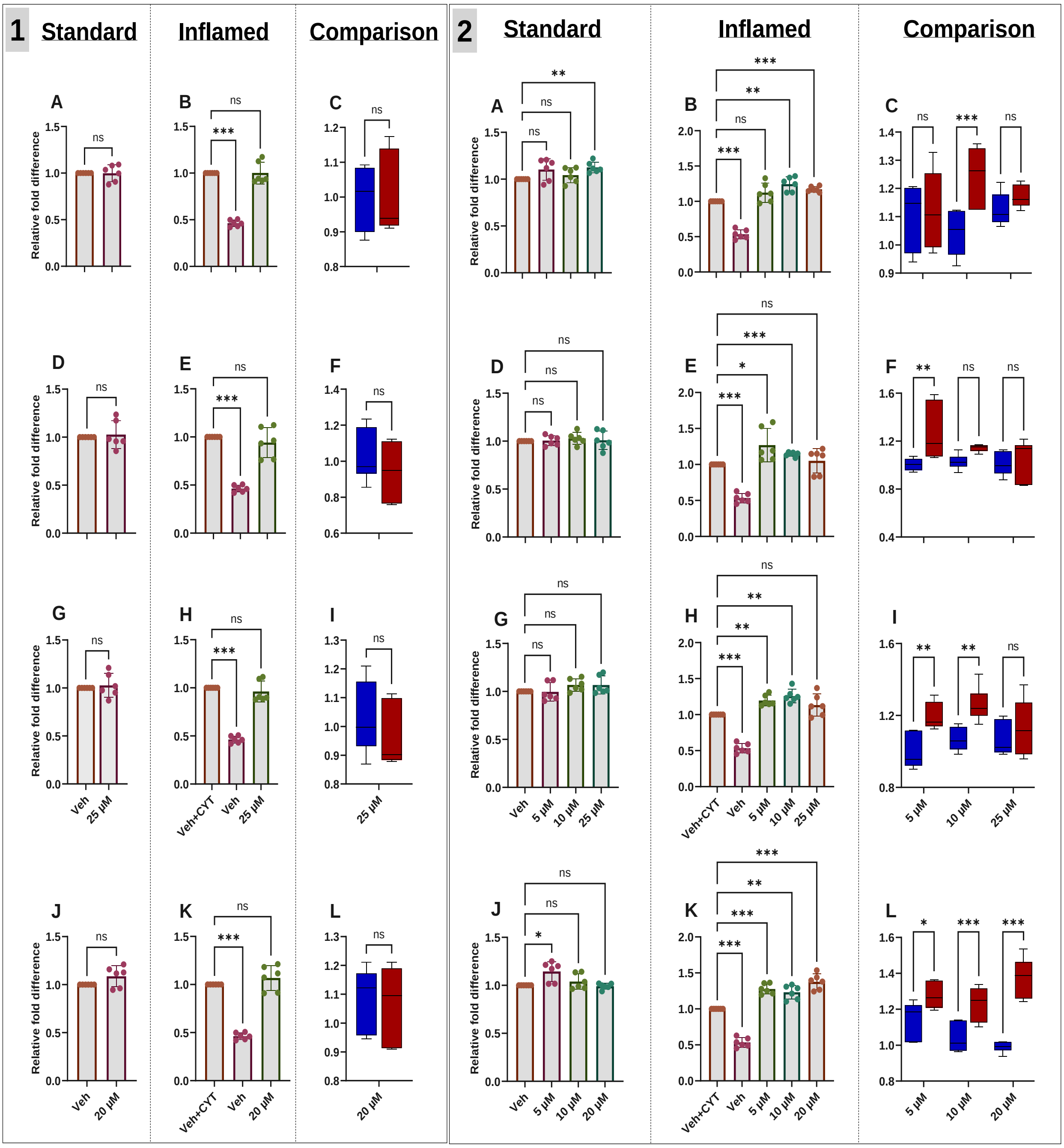

Figure S5: Acute and chronic effect of phytochemicals on ACE2 protein in standard and inflamed Caco-2/TC7 cells. The acute (Panels 1) and chronic (Panels 2) effects of genistein (A-C), apigenin (D-F), artemisinin (G-I) and sulforaphane (J-L) on ACE2 protein were measured in Caco-2/TC7 cells, cultured in standard (first column of each Panel) or inflammatory (second column of each Panel) conditions. Cells were treated with the phytochemicals (5, 10 or 25 µM genistein, apigenin or artemisinin, or 5, 10 or 20 µM

sulforaphane) or 0.1% (v/v) DMSO vehicle control (Veh) for either 4 h (acute) or 60 h (chronic), with or without exposure to the cytokine cocktail of IL-1β (25 ng/mL) and TNF-α (50 ng/mL) for 72 h (+CYT), as outlined in **Figure 4A**. A comparison of the effect of each compound in the standard (blue) and inflammatory (red) condition is shown in the third column of each Panel. For protein, cells from three biological passages were lysed and ACE2 protein was assayed in duplicate using ELISA, corrected to total protein, and presented as mean fold changes compared to the control (with or without cytokine exposure accordingly). Data are mean ± SD (n/N = 6/3). Significant differences were determined as follows. Panels 1: standard and comparison - unpaired t-test with Welch's correction; inflamed - one-way ANOVA with Fisher's LSD multiple comparisons test. Panels 2: standard and inflamed - one-way ANOVA with Fisher's LSD multiple comparisons test; comparison - two-way ANOVA with Fisher's LSD multiple comparisons test. ns - not significant, *$P < 0.05$, **$P < 0.01$, ***$P < 0.001$.

References

1. Tito A, Colantuono A, Pirone L, Pedone E, Intartaglia D, Giamundo G, et al. Pomegranate Peel Extract as an Inhibitor of SARS-CoV-2 Spike Binding to Human ACE2 Receptor (in vitro): A Promising Source of Novel Antiviral Drugs. Front Chem. 2021;9: 1–11. doi:10.3389/fchem.2021.638187

2. Liu H, Ye F, Sun Q, Liang H, Li C, Li S, et al. Scutellaria baicalensis extract and baicalein inhibit replication of SARS-CoV-2 and its 3C-like protease in vitro. J Enzyme Inhib Med Chem. 2021;36: 497–503. doi:10.1080/14756366.2021.1873977

3. Takahashi Y, Lavigne JA, Hursting SD, Chandramouli GVR, Perkins SN, Barrett JC, et al. Using DNA microarray analyses to elucidate the effects of genistein in androgen-responsive prostate cancer cells: Identification of novel targets. Mol Carcinog. 2004;41: 108–119. doi:10.1002/mc.20045

4. Takahashi Y, Lavigne JA, Hursting SD, Chandramouli GVR, Perkins SN, Kim YS, et al. Molecular signatures of soy-derived phytochemicals in androgen-responsive prostate cancer cells: A comparison study using DNA microarray. Mol Carcinog. 2006;45: 943–956. doi:10.1002/mc.20247

5. Kesic MJ, Simmons SO, Bauer R, Jaspers I. Nrf2 expression modifies influenza A entry and replication in nasal epithelial cells. Free Radic Biol Med. 2011;51: 444–453. doi:10.1016/j.freeradbiomed.2011.04.027

6. Gendrot M, Duflot I, Boxberger M, Delandre O, Jardot P, Le Bideau M, et al. Antimalarial artemisinin-based combination therapies (ACT) and COVID-19 in Africa: In vitro inhibition of SARS-CoV-2 replication by mefloquine-artesunate. International Journal of Infectious Diseases. 2020;99: 437–440. doi:10.1016/j.ijid.2020.08.032

7. Nunes JJ, Pandey SK, Yadav A, Goel S, Ateeq B. Targeting NF-kappa B Signaling by Artesunate Restores Sensitivity of Castrate-Resistant Prostate Cancer Cells to Antiandrogens. Neoplasia. 2017;19: 333–345. doi:10.1016/j.neo.2017.02.002

8. Horne JR, Vohl M-C. Biological plausibility for interactions between dietary fat, resveratrol, ACE2 , and SARS-CoV illness severity. American Journal of Physiology-Endocrinology and Metabolism. 2020;318: E830–E833. doi:10.1152/ajpendo.00150.2020

9. Jena AB, Kanungo N, Nayak V, Chainy GBN, Dandapat J. Catechin and curcumin interact with S protein of SARS-CoV2 and ACE2 of human cell membrane: insights from computational studies. Sci Rep. 2021;11: 2043. doi:10.1038/s41598-021-81462-7

10. Kaul R, Paul P, Kumar S, Büsselberg D, Dwivedi VD, Chaari A. Promising Antiviral Activities of Natural Flavonoids against SARS-CoV-2 Targets: Systematic Review. Int J Mol Sci. 2021;22: 11069. doi:10.3390/ijms222011069

11. Antonio ADS, Wiedemann LSM, Veiga-Junior VF. Natural products' role against COVID-19. RSC Adv. 2020;10: 23379–23393. doi:10.1039/D0RA03774E

12. Kumar A, Choudhir G, Shukla SK, Sharma M, Tyagi P, Bhushan A, et al. Identification of phytochemical inhibitors against main protease of COVID-19 using molecular modeling approaches. J Biomol Struct Dyn. 2021;39: 3760–3770. doi:10.1080/07391102.2020.1772112

13. Group RC. Dexamethasone in Hospitalized Patients with Covid-19. New England Journal of Medicine. 2021;384: 693–704. doi:10.1056/NEJMoa2021436

14. Sato T, Suzuki T, Watanabe H, Kadowaki A, Fukamizu A, Liu PP, et al. Apelin is a positive regulator of ACE2 in failing hearts. Journal of Clinical Investigation. 2013;123: 5203–5211. doi:10.1172/JCI69608

15. Ojeda NB, Grigore D, Robertson EB, Alexander BT. Estrogen Protects Against Increased Blood Pressure in Postpubertal Female Growth Restricted Offspring. Hypertension. 2007;50: 679–685. doi:10.1161/HYPERTENSIONAHA.107.091785

16. Lucas JM, Heinlein C, Kim T, Hernandez SA, Malik MS, True LD, et al. The Androgen-Regulated Protease TMPRSS2 Activates a Proteolytic Cascade Involving Components of the Tumor Microenvironment and Promotes Prostate Cancer Metastasis. Cancer Discov. 2014;4: 1310–1325. doi:10.1158/2159-8290.CD-13-1010

17. Tikoo K, Patel G, Kumar S, Karpe PA, Sanghavi M, Malek V, et al. Tissue specific up regulation of ACE2 in rabbit model of atherosclerosis by atorvastatin: Role of epigenetic histone modifications. Biochem Pharmacol. 2015;93: 343–351. doi:10.1016/j.bcp.2014.11.013

18. Leach DA, Mohr A, Giotis ES, Cil E, Isac AM, Yates LL, et al. The antiandrogen enzalutamide downregulates TMPRSS2 and reduces cellular entry of SARS-CoV-2 in human lung cells. Nat Commun. 2021;12: 4068. doi:10.1038/s41467-021-24342-y

19. Wong C, Kelce WR, Sar M, Wilson EM. Androgen receptor antagonist versus agonist activities of the fungicide vinclozolin relative to hydroxyflutamide. J Biol Chem. 1995;270: 19998–20003. doi:10.1074/jbc.270.34.19998

20. Saheb Sharif-Askari N, Saheb Sharif-Askari F, Alabed M, Tayoun AA, Loney T, Uddin M, et al. Effect of Common Medications on the Expression of SARS-CoV-2 Entry Receptors in Kidney Tissue. Clin Transl Sci. 2020;13: 1048–1054. doi:10.1111/cts.12862

21. Cai G, Bossé Y, Xiao F, Kheradmand F, Amos CI. Tobacco Smoking Increases the Lung Gene Expression of ACE2, the Receptor of SARS-CoV-2. Am J Respir Crit Care Med. 2020;201: 1557–1559. doi:10.1164/rccm.202003-0693LE

22. Khan MK, Ahmad K, Hassan S, Imran M, Ahmad N, Xu C. Effect of novel technologies on polyphenols during food processing. Innovative Food Science & Emerging Technologies. 2018;45: 361–381. doi:10.1016/j.ifset.2017.12.006

23. Khatiwada S, Subedi A. A Mechanistic Link Between Selenium and Coronavirus Disease 2019 (COVID-19). Curr Nutr Rep. 2021;10: 125–136. doi:10.1007/s13668-021-00354-4

24. Farooqi AA. Therapeutic Effect of Epigallocatechin-3-gallate (EGCG) and Silibinin on ATM Dynamics in Prostate Cancer Cell Line LNCaP. World J Oncol. 2010;1: 242–246. doi:10.4021/wjon248w

25. Kim HR, Kim WK, Ha AW. Effects of Phytochemicals on Blood Pressure and Neuroprotection Mediated Via Brain Renin-Angiotensin System. Nutrients. 2019;11: 2761. doi:10.3390/nu11112761
